# New light on the use of *Theobroma cacao* by Late Classic Maya

**DOI:** 10.1073/pnas.2121821119

**Published:** 2022-09-26

**Authors:** Anabel Ford, Ann Williams, Mattanjah S. de Vries

**Affiliations:** ^a^Institute of Social Behavior and Economic Research/MesoAmerican Research Center, University of California, Santa Barbara, CA 93106-2150;; ^b^Department of Chemistry and Biochemistry, ISBER/MesoAmerican Research Center, University of California, Santa Barbara, CA 93106-9510

**Keywords:** Maya, cacao, archaeometry

## Abstract

Cacao seeds, *Theobroma cacao*, provide the basis for a ceremonially important Mesoamerican food. Past efforts to identify cacao in ceramics focused on highly decorative vessel forms associated with elite ceremonial contexts, creating assumptions as to how cacao was distributed and who could access it. This study examines 54 archaeological ceramic sherds from El Pilar (Belize/Guatemala) of Late Classic (600 to 900 CE) residential and civic contexts representing a cross-section of ancient Maya inhabitants. Identification of cacao in ancient sherds has depended on the general presence of theobromine; we used the discrete presence of theophylline, a unique key biomarker for cacao in the region. Analysis was done by grinding off all outside surfaces to reduce contamination, pulverizing the inner clay matrix, extracting absorbed molecules, and concentrating the extractions. In order to obtain especially high selectivity and low limits of detection, our study utilized the technique of resonance-enhanced multiphoton ionization coupled with laser-desorption jet-cooling mass spectrometry. This technique isolates molecules in the cold gas phase where they can be selectively ionized through a resonant two-photon process. Of the sherds analyzed, 30 samples (56%) were found to contain significant amounts of theophylline and thus test positive for cacao. Importantly, cacao is present in all contexts, common to all Maya residents near and far from centers.

## Significance

In order to address the distribution of and access to cacao, 54 sherds from Late Classic Period Maya residential and civic contexts around El Pilar (Belize/Guatemala) were tested for the presence of cacao. Positive identification of cacao requires that the technique of laser mass spectrometry detect a significant amount of the key biomarker of theophylline to signify cacao. Results show that cacao was culturally significant and widespread and found in civic and residential units regardless of size and location.

**Fig. 1.**
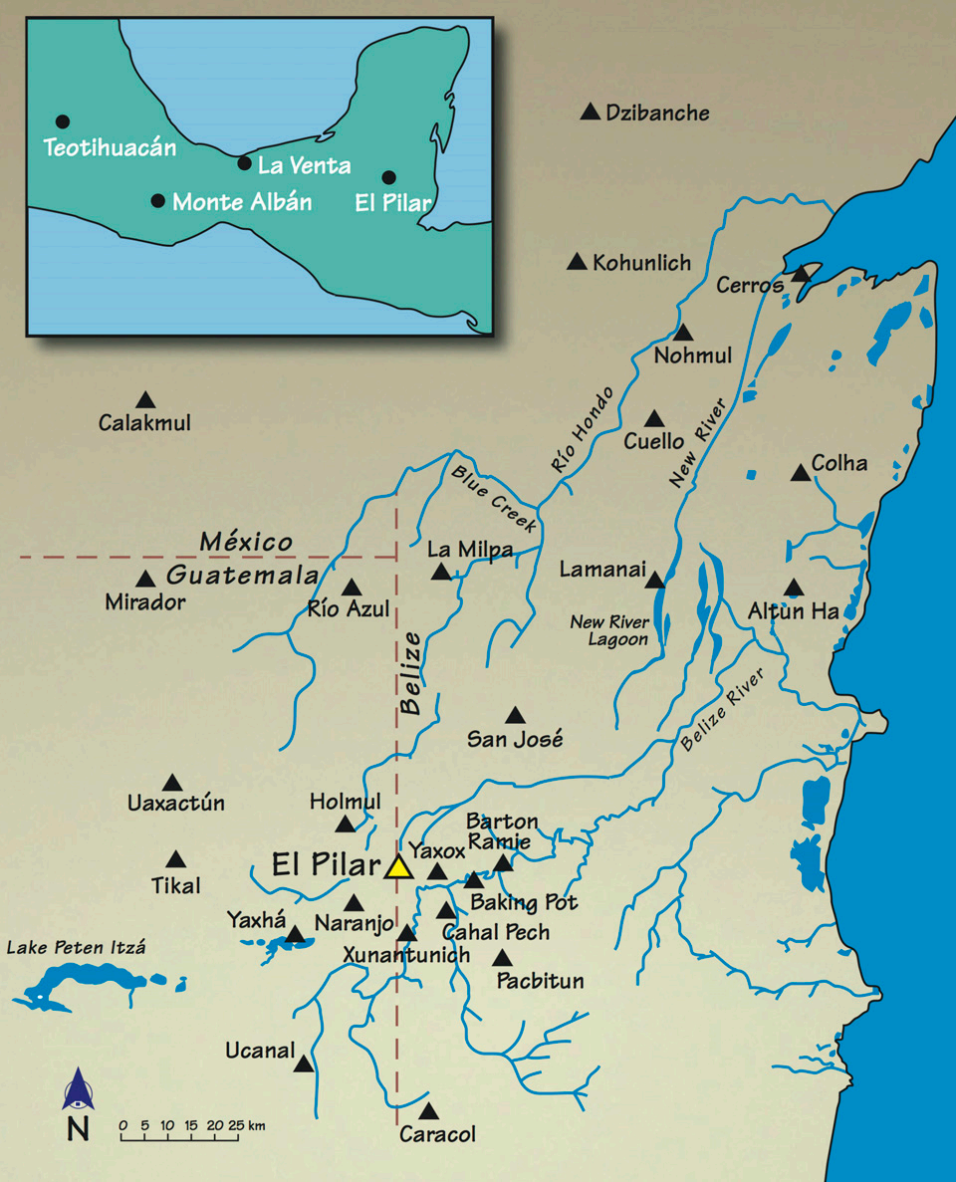
Mesoamerica and the Maya Lowlands with El Pilar at the ecotone between the interior and the Belize River, with other major civic centers Indicated. Image credit: MesoAmerican Research Center.

Cacao, known as the money that grew on trees, was brought to the world stage by Mesoamericans and the Maya (Fig. 1) (1, 2). The historical background and prehistory of cacao, particularly for the Maya, has been imbued with ceremony and luxury. Studies of glamourous drinking vases, prominent among Late Classic Maya (600 to 900 CE) ceramic vessels highlight imagery of gift giving, ritual, royal prestige, and control. Building on the assumption that ancient Maya royalty controlled cacao disregards the notion that farmer households are those that grow cacao with direct access to their products, and cacao’s archaeobotanical presence in residential contexts supports this (3–9). Consequently, the search for cacao residues has targeted regal contexts, but what of the general populace? Our study looks at vessel belongings from both civic and residential contexts across the greater El Pilar area to test the exclusivity of cacao use.

In recent decades, studies of cacao residues from ancient ceramics have employed methylxanthine biomarkers—caffeine, theobromine, and theophylline—ushering in a new means of addressing the use of cacao in prehistory. Chemical residue analyses have focused on vessels from elite Maya burials and caches, suggesting cacao represented wealth and power (10–12). Cacao residues have been documented in special vessels from the Late Preclassic, circa 300 BCE (13), identified in a famous Early Classic vessel from Rio Azul (14), and recognized in Late Classic vases (10, 15).

Cacao residues from Maya samples have been associated with special and specific archaeological settings at civic centers (10, 15). As the emphasis of residue studies in the Maya area has been on vessels from sumptuary settings, evidence of the presence, access to, and distribution of the consumption of cacao in general residential settings has not been tested. We close this gap by examining a variety of Late Classic Maya vessels from civic and residential contexts of the El Pilar area. For continuity we include vessels from centers, while featuring household contexts to address the consumption of cacao.

**Fig. 2.**
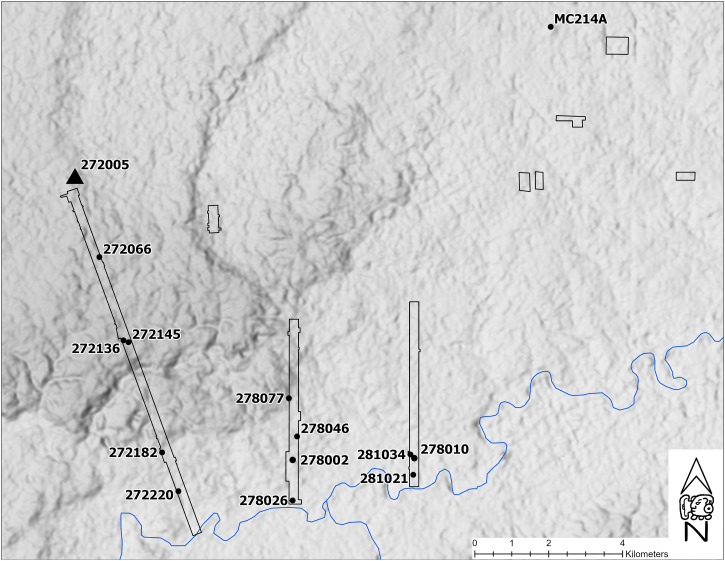
El Pilar Belize River area-study sites, including centers El Pilar (272005), Yaxox (278002), and center of Bacab Na (281-10). Image credit: MesoAmerican Research Center.

The examination of Late Classic Maya vessels from households around the general El Pilar area of the central Maya lowlands (Figs. 1 and 2) provides an excellent example case based on local test and full-scale excavations at Maya residential units. What is cacao consumption among the Maya populace? Is consumption restricted to higher ranking houses? Are farmers who might grow cacao, like those of Ceren (4) also consuming cacao? Only a full chemical analysis of the presence of methylxanthine biomarkers in ceramic vessel samples from a wide selection of contexts, including the civic and residential components of Maya society, can answer these questions.

## Methylxanthines and Cacao.

The methylxanthines (*SI Appendix*, Fig. S1) caffeine (C), theobromine (TB), and theophylline (TP) have been found worldwide in 100 species, including 13 orders of the plant kingdom. Most of these plants have only trace amounts of these compounds. TB and C are relatively common, while TP is rare. The few plants that do have concentrations of all three biomarkers have greater amounts of both TB and C compared with TP (16, 17). According to the Dr. Duke’s Phytochemical and Ethnobotanical Databases (https://phytochem.nal.usda.gov), TP is identified today in seven plants: *Camellia sinensis*, *Coffea arabica*, *Ilex paraguariensis*, *Paullinia cupana*, *Theobroma angustifolium*, *Theobroma bicolor*, and *Theobroma cacao.* This finding is corroborated by archaeological literature (18, 19). We used the US Department of Agriculture GRIN-Global database to determine the regional location of each of these plants (https://npgsweb.ars-grin.gov/gringlobal) to understand the implications of the identification of TP, keeping in mind that, as with many plants, their native distribution now is wider than before the great Columbian exchange 500 y ago. Our database search makes it clear that *T. cacao* is unique in Mesoamerica for TP, giving us confidence that any TP detected in our samples will be from cacao rather than other natural sources.

To date, relying on TB as a biomarker for clear identification of cacao has been confounded by the fact that TB, together with C, is a signature for two different New World stimulants: cacao, *T. cacao*, and holly black drink, *Ilex vomitoria* and *Ilex cassine* (20). This challenge calls for the use of a distinct methylxanthine biomarker to separate out cacao residue from black drink residue. In the context of the Mesoamerica, TP is unique to cacao and thus can present a key to its identification (21). We used a precision technique of two-step laser mass spectrometry (L2MS) for the detection of all three methylxanthine biomarkers, TB, C, and TP, in order to investigate the presence of cacao in Late Classic Maya vessels from El Pilar.

## Late Classic Maya Samples from El Pilar.

El Pilar is a major Maya center in the interior zone of Tikal at the ecotone of the Belize Valley. The Maya lowlands of El Pilar comprise distinct landform zones covering around 1,300 km^2^ (Fig. 2). El Pilar, a major Maya center in Belize and Guatemala (22–27), dominated the eastern Maya lowlands of the upper Belize River area in the Late Classic period, where toponymic references to cacao are recognized (28). Built over centuries and extending across circa 100 ha of monumental civic architecture, El Pilar is situated in the well-drained upland ridges (29–31) at an ecotone where uplands meet valley, providing a varied domesticated landscape (32). The uplands form 40% of the Belize River area, where settlements were concentrated in zones well-suited to general food production (31). The valley forms about 5% of the area (33) and is characterized by evenly dispersed settlement and minor civic centers with excellent farmlands. Colonial reports indicate cacao production at the time of the conquest (34). The intervening foothills rising from the valley form 55% of the area, with minor centers, dispersed small settlements, and poorly drained soils ill-suited for concentrated agricultural pursuits. This complex environmental setting furnished the mosaic landscape that provisioned the ancient Maya (35).

**Fig. 3.**
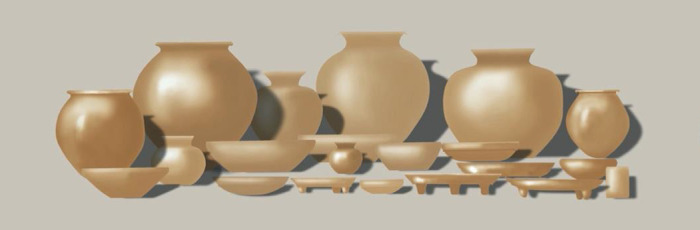
Late Classic vessel assemblage composed of 5% vases, 35% jars, 40% bowls, and 20% plates. Image credit: MesoAmerican Research Center.

Our study focuses on ceramic collections from the regional surveys of surface collection and test excavations, as well as the focus construction and midden excavations of residential units and civic centers of the El Pilar area (22, 24, 30, 33, 36–48). Collections were washed, sorted, and cataloged in the field and then bagged and transported to storage at the University of California, Santa Barbara (UCSB). The materials selected for the residue examination include a total of 54 Late Classic vessels, of which 13 were analyzed () in our preliminary study (Table 1) and 41 examined in the present study. The items used in our sample favor the vases, as an example of drinking vessels that were presumed to be used for consuming beverages, and include general purpose storage jars, mixing bowls, and serving plates that are common Late Classic ceramic vessels (Fig. 3). Samples were designed to examine areas where cacao would accumulate: bases were primary and rims were secondary. The combined selection incorporates vessels from major and minor centers and, importantly, large, medium, and small residential units (49–51) in the Belize River area both near and far from the major center, El Pilar.

**Table 1.** Analytical results of archaeological samples in this study from the El Pilar area

No.	Catalog no.	Site	Form	TB	C	TP	TP/C ratio
1	10157	MC214A	Bowl rim	0.38	**2.78**	1.16	—
2	1313	278-077	Plate rim	0.7	**3.72**	0.99	—
3	1016A	Bacab Na	Plate rim	0.52	**1.28**	**1.85**	0.84
4	1307	278-077	Plate rim	0.3	**5.8**	**2.04**	0.2
5	2005	272-32	Plate base	0.3	**3.48**	0.96	—
6	14387A	278-066	Vase base	0.2	0.15	0.79	—
7	14387AW	278-066	Vase base	0.52	**2.06**	**2.78**	0.79
8	14387AS	278-066	Vase base	0.355	**2.47**	**1.72**	0.41
9	14387AQ	278-066	Vase base	0.99	**10.60**	1.4	—
10	16484 HM	272-182	Vase base	0.17	0.18	0.15	—
11	15497AH	272-182	Vase base	0.905	**3.48**	**3.13**	0.52
12	16499 AI	272-182	Vase base	0.32	0.88	**2.67**	—
13	16484HI	272-182	Vase base	0.77	1.11	**3.41**	—
14	16484HE	272-182	Vase base	0.79	0.36	**2.28**	—
15	16527CJ	272-182	Vase base	1.09	0.83	**3.79**	—
16	16445H	272-182	Vase base	0.52	**2.24**	**2.05**	0.53
17	16484HW	272-182	Vase base	0.815	1.24	**1.71**	—
18	16531O	272-136	Vase base	0.24	0.47	**6.95**	—
19	16581A	272-136	Vase base	0.32	0.89	1.48	—
20	13133A	281-21	Vase base	0.01	0	1.12	—
21	13171H	281-21	Vase base	0.42	0	0.98	—
22	13171E	281-21	Vase base	0.43	0.27	0.65	—
23	13184Q	281-21	Vase base	0.28	0.19	0.62	—
24	16425N	272-145	Vase base	0.37	0	1.03	—
25	16589A	272-145	Vase base	0.59	**4.23**	0.87	—
26	16379H	272-145	Vase base	0.42	**2.69**	1.19	—
27	16535AA	272-145	Vase base	0.64	**1.71**	**3.32**	1.13
28	16578H	272-145	Vase base	1.15	**1.9**	1.1	—
29	16425N	272-145	Vase base	0.58	**2.69**	1.3	—
30	14230AL	272-220	Vase base	0.56	0.47	**1.75**	—
31	14230ALL	272-220	Vase base	0.28	**1.64**	**7.58**	2.69
32	16170FE	272-145	Bowl rim	0.215	**3.77**	1.11	—
33	3656	272-198	Vase tripod	1.24	**3.77**	**1.71**	0.26
34	16170FT	272-145	Bowl rim	0.69	**2**	**1.57**	0.46
35	16170 FF	272-145	Bowl rim	0.5	**2.62**	1.23	—
36	4798A	El Pilar	Jar rim	1.37	**5.79**	**3.65**	0.37
37	16359A	272-145	Jar rim	0.16	**13.36**	0.85	—
38	16427A	272-145	Jar rim	0.38	**10.29**	0.7	—
39	4684	El Pilar	Jar rim	0.42	**11.91**	0.52	—
40	4675	El Pilar	Jar rim	0.69	**7.56**	0.07	—
41	4751	El Pilar	Jar rim	0.98	**10.3**	0.62	—

Boldface indicates that the signal is higher than S/N value of 1.5.

Expanding on the pilot sample of Late Classic Maya vessels—of which 12 of 13 vases were determined to contain cacao residue due to the presence of TP (21)—the present work incorporates wider residential settings alongside additional vessel forms to address questions of cacao consumption. This study reports on the presence of cacao based on residue analysis results from household belongings and centers in the three characteristic landform zones: the densely occupied upland ridges, the low-density transitional foothills, and the moderate density alluvial valley. Importantly, our results demonstrate that the stimulant, cacao, was widely consumed in the royal as well as residential settings.

## Background of Cacao Identification in the New World.

The use of biomarkers has provided a crucial means to identify food and beverage residues in archaeological artifacts. Recognition of cacao through biomarkers began in 1989 with the identification of TB in a decorative vessel from Rio Azul by Hurst et al. (52). The search for cacao using the biomarker TB then rapidly spread to other areas. Investigations moved from Mesoamerica (53–54) to the greater American southwest and southeast (20, 55–57). The finding of TB in more archaeological sites in different locations led to new questions. Was cacao really distributed so widely, or were measurements of biomarkers leading to false positives (19)? In addition, complexities arose in identifying cacao in North America because black drink (20, 56), another New World stimulant, contains the biomarkers TB and C. In other words, is the detection of TB alone sufficient for a positive attribution of cacao?

Another means of identifying cacao is the ratio of the three biomarkers TB, C, and TP. Previous archaeological cacao residue studies have reported that unprocessed raw cacao beans have a ratio of TB/C of 1:2 to 11. This very large range is due to the fact that there are three different varieties of *T. cacao*: Criollo, Forastero, and Trinitario (58). Criollo, the ancestral variety grown by Mesoamericans and the Maya prior to the conquest (59, 60), has a narrow TB/C ratio of 1:2 to 3 (61). The North American black drink is derived from two different holly species. *I. vomitoria* has a TB/C ratio of 1:5 and *I. cassine* has a ratio of 1:2 (56). The ratio in *I. cassine* is similar to that of ancestral cacao species, yet *I. cassine* has a restricted geographic distribution focused around the Florida peninsula, while *I. vomitoria* has a wider distribution across the greater southeastern United States (56). Several studies have analyzed samples to distinguish cacao from black drink using these ratios and attributing results to cacao if there was more TB than C or if TP was present (20, 21, 57, 62).

There are several problems when using biomarker ratios for cacao attribution. Many factors can affect the ratios. During the period of vessel use, biomarkers may unequally adsorb into the clay matrix and there is an unknown history of mixed uses that could further complicate the interpretation of ratios (11). After discard, biomarkers could leach out at different rates, depending on solubility and the nature of the archaeological deposit. Compounding the issue, Washburn et al. has shown that the extraction procedure can unevenly remove the biomarkers (18, 63).

While identifying TP is clearly significant, most traditional analytical techniques are not sufficiently sensitive to detect TP, reporting concentrations at or close to the limits-of-detection (LOD). We present a technique that is especially sensitive for TP, elevating the detection of TP to a key biomarker for cacao in Mesoamerica.

## Results

We established calibration curves for the analytes C, TB, and TP (*SI Appendix*, Fig. S2). For each compound, we measured standard samples of 39 pg, 78 pg, 780 pg, 1.95 ng, and 2.9 ng. Each biomarker has a different response due to different two-photon ionization efficiencies. From the slopes of these curves, we derived the relative sensitivity for TB, C, and TP as 1:2.7:4.4. The LOD is based on a signal-to-noise (S/N) level of 3 SDs in the background signal, corresponding to a signal of 1.5. Using the calibration curves, this signal translates to an LOD of 228 pg for TB, 85 pg for C, and 52 pg for TP.

Archaeological samples were selected for their relevance to the question of cacao distribution among the Maya. Comparable analytical steps were used in both our initial experimental study and the intense study presented here. Each archaeological sample was prudently analyzed with three replicates. For each replicate, 12 iterations of signal were collected and averaged. Additionally, three shots of background were collected, obtained by blocking the desorption laser. The final reported signal of each archaeological sample is the overall calculated average of the three replicates.

Studies have shown that cotton balls, sandpaper, and dust all can contain methylxanthine contaminants (19, 57, 62), so we carefully analyzed blank substrates with five replicates each to address this issue. Blanks consisted of a graphite bar with carbon tape and graphite on it. The signals for TB, C, and TP were 0.15, 0.6, and 1.11, respectively, all below the 1.5 value of 3 SDs in the background signal. Of the 41 archaeological samples, signal values were within the background noise level for TB in all samples, for C in 16 samples, and for TP in 23 samples. Eight of the samples were within the background noise level for all three compounds. These observations provide confidence that our laboratory analysis does not add contamination to the samples at levels exceeding our LOD.

Our analytical technique requires precise alignment, so we used a concentration standard of 0.78 ng for periodic calibration to ensure the reproducibility of our measurements.

Table 1 lists the signal obtained for each molecule. Entries are boldfaced when the signal exceeded the background S/N value of 1.5, our criterion to consider the sample as positive for the biomarker. The TP/C ratios are shown for the samples where both methylxanthines are positive.

For the 41 samples of our intensive study, no TB signal exceeded the background, thus we could not detect TB above our LOD of 228 pg. Twenty-five samples were positive for C, which has a much lower LOD of 85 pg. Eighteen samples tested positive for TP, with lowest LOD of 52 pg, of which 11 were also positive for C. Adopting the standard that detectable presence of TP is the best means to identify the presence of cacao in New World samples, our results indicate that 18 samples tested positive for cacao.

In Table 1, TP/C ratios for the 11 samples with significant signal from both compounds are listed. These ratios range from 0.2 to 2.69, much lower than other reported TP/C ratios of 6.12, 7.31, 7.34, and 26.9 (20, 57, 62). Somewhere between the raw cacao bean and the archaeological sample analysis, there are processes affecting the ratio, rendering this variable unreliable.

## Discussion

### Archaeological Implications.

The search for evidence of cacao residue in archaeological vessels has been propelled by the narrative of its high prestige, suggesting that consumption was restricted (11–16) and used to maintain positions of privilege and power (15).

Our investigation of belongings associated with residential units and civic centers in the El Pilar area is unique in addressing the wider context of cacao consumption among the Late Classic Maya. Sample vessels were analyzed in the two phases. Our initial test of 13 vases was designed to determine if cacao could be detected (21). With our technique validated, we expanded the sample to investigate cacao distribution by selecting an additional 41 vessels from large, medium, and small residential units in the upland ridges, the transitional foothills, and the alluvial Belize River valley, where cacao was documented at the time of the conquest (34). Sampled civic centers include the major center El Pilar, the minor centers Bacab Na, Yaxox, and MC214A (*SI Appendix*, Table S1). Together, the 54 samples provide a window for viewing the importance of cacao among the ancient Maya (*SI Appendix*, Table S1).

The historical record of cacao consumption begins with Spanish accounts. Vases, sometimes referred to as cups (8), have been identified as vessels suitable for beverages (53, 60, 64–66). This is substantiated by iconographic and textual information from the Classic Maya (12). In our sample selection (*SI Appendix*, Table S2), we gave preference to vases (*n* = 40), the iconic drinking vessels, and also included storage jars (*n* = 6), mixing bowls (*n* = 4), and serving plates (*n* = 4). Sampling procedures incorporated the nature of the sites from which these belongings were collected, representing 10 residential units and 4 civic centers (Fig. 2 and Table 2). Our complete dataset contains samples of all common Late Classic vessel forms (Fig. 3 and *SI Appendix*, Table S2), and examples of each form evince the presence of cacao biomarkers.

**Table 2.** Sample cacao determination by ancient Maya site, vessel form, landform, site type, and context

Catalog no.	Site	Form	Cacao	Landform	Site type	Context
2005	272-32	Plate base	Negative	Uplands	Small RU	M
16531O	272-136	Vase base	Positive	Uplands	Large RU	C
16581A	272-136	Vase base	Negative	Uplands	Large RU	C
16425N	272-145	Vase base	Negative	Uplands	Large RU	C
16589A	272-145	Vase base	Negative	Uplands	Large RU	C
16379H	272-145	Vase base	Negative	Uplands	Large RU	C
16535AA	272-145	Vase base	Positive	Uplands	Large RU	C
16425N	272-145	Vase base	Negative	Uplands	Large RU	C
16170FE	272-145	Bowl rim	Negative	Uplands	Large RU	C
16170FT	272-145	Bowl rim	Positive	Uplands	Large RU	C
16170 FF	272-145	Bowl rim	Negative	Uplands	Large RU	C
16359A	272-145	Jar rim	Negative	Uplands	Large RU	C
16427A	272-145	Jar rim	Negative	Uplands	Large RU	C
16463G	272-145	Vase base	Positive	Uplands	Large RU	C
16535Z	272-145	Vase base	Positive	Uplands	Large RU	C
16359AB	272-145	Vase base	Positive	Uplands	Large RU	C
16578H	272-145	Vase base	Negative	Uplands	Large RU	C
16484 HM	272-182	Vase base	Negative	Foothills	Small RU	C
15497AH	272-182	Vase base	Positive	Foothills	Small RU	C
16499 AI	272-182	Vase base	Positive	Foothills	Small RU	C
16484HI	272-182	Vase base	Positive	Foothills	Small RU	C
16484HE	272-182	Vase base	Positive	Foothills	Small RU	C
16527CJ	272-182	Vase base	Positive	Foothills	Small RU	C
16445H	272-182	Vase base	Positive	Foothills	Small RU	C
16484HW	272-182	Vase base	Positive	Foothills	Small RU	C
3656	272-198	Vase tripod	Positive	Uplands	Large RU	M
14230AL	272-220	Vase base	Positive	Foothills	Medium RU	C
14230ALL	272-220	Vase base	Positive	Foothills	Medium RU	C
14387A	278-066	Vase base	Negative	Foothills	Small RU	M
14387AS	278-066	Vase base	Positive	Foothills	Small RU	M
14387AW	278-066	Vase base	Positive	Foothills	Small RU	M
14387AQ	278-066	Vase base	Negative	Foothills	Small RU	M
1313	278-077	Plate rim	Negative	Foothills	Small RU	M
1307	278-077	Plate rim	Positive	Foothills	Small RU	M
2009	278-39	Vase base	Positive	Foothills	Small RU	M
14321V	278-26	Vase base	Positive	Valley	Small RU	C
14328K	278-26	Vase base	Positive	Valley	Small RU	C
143325U	278-26	Vase base	Positive	Valley	Small RU	C
14320M	278-26	Vase base	Negative	Valley	Small RU	C
14319O	278-26	Vase base	Positive	Valley	Small RU	C
14342V	278-26	Vase base	Positive	Valley	Small RU	C
13133A	281-21	Vase base	Negative	Valley	Medium RU	C
13171H	281-21	Vase base	Negative	Valley	Medium RU	C
13171E	281-21	Vase base	Negative	Valley	Medium RU	C
13184Q	281-21	Vase base	Negative	Valley	Medium RU	C
13394AO	281-21	Vase base	Positive	Valley	Medium RU	C
578	281-34	Vase base	Positive	Valley	Medium RU	M
1016A	Bacab Na	Plate rim	Positive	Valley	Minor center	C
4798A	El Pilar	Jar rim	Positive	Uplands	Major center	C
4684	El Pilar	Jar rim	Negative	Uplands	Major center	C
4675	El Pilar	Jar rim	Negative	Uplands	Major center	C
4751	El Pilar	Jar rim	Negative	Uplands	Major center	C
10157	MC214A*	Bowl rim	Negative	Foothills	Minor center	C
4209	Yaxox	Vase base	Positive	Foothills	Minor center	C

C, construction; M, midden; RU, residential unit.

^*^Located 10 km east of El Pilar.

**Fig. 4.**
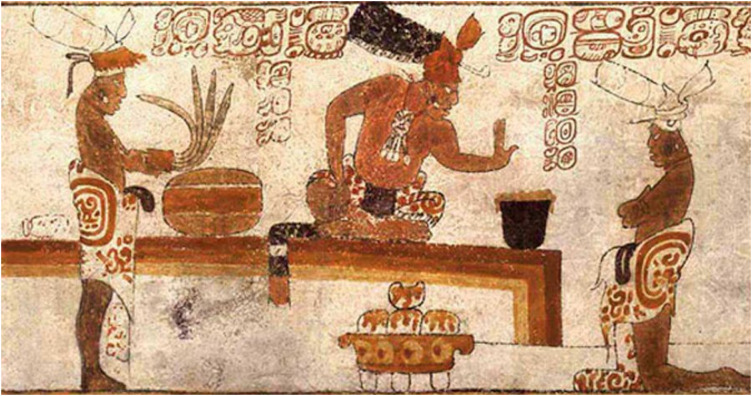
Painted cylinder vase depicting a foaming drink in a vase and tamales in a plate. Image credit: Justin Kerr, mayavase.com, Kerr number: 6418 (88).

While vases dominated the study (74%), samples of all domestic ceramic forms allow us to investigate the consumption of cacao by the Late Classic Maya. Interestingly, positive designation for cacao was determined for all forms, not just the designated drinking vessels. Chemical traces of cacao were present in bowls for mixing, jars for storage, and plates for serving (*SI Appendix*, Table S2). Indeed, there are images on vases (Fig. 4) that show tamales with a topping; are these picturing *mole de cacao*?

This study emphasized residential settings, yet included examples from civic-ceremonial places as well, demonstrating a significantly wider role of cacao consumption than has been assumed. We conclude that cacao biomarkers are common in many Late Classic contexts, and can be recognized in all basic domestic vessel forms, across every landform in the El Pilar area, in residential units of every status and, of course, in civic centers (*SI Appendix*, Table S3). If cacao was employed in prestigious rituals, such events were shared by all ranks of society.

These results dispel any doubt as to the importance and inclusiveness of cacao consumption among the Late Classic Maya. That cacao is generally available does not diminish its value but contextualizes its formal and ceremonial importance as a cultural phenomenon that experienced wide participation by the populace. Well beyond the elite ritual civic-ceremonial realm, we interpret the identification of cacao in vessels belonging to people of all walks of life as confirmation that cacao’s prestige was consumed by all in Maya society. If cacao was “tightly bound together with alliance formation and socioeconomic enrichment” (12), then cacao’s role was universal in Late Classic Maya society and consuming cacao was essential for prestige and privilege among the population as a whole. Formal celebrations recognized in regal formats were cultural features that must have included everyone. It may well be that rituals occasioned reciprocal relationships between elites in the community, such as was recorded with balché use (67), was appreciated widely. The proposed events involving cacao consumption were more common than originally anticipated. With collaborative studies such as ours, where chemistry and archaeology meet, we now can show that cacao consumption was shared among all members of ancient Maya communities.

### Conclusions.

New light is cast on the ancient Maya by a close examination of the nature of cacao residue in their vessels. We have used the L2MS technique to unite very high sensitivity and specificity through the combination of high-resolution optical spectroscopy and MS for identifying cacao. Our positive identification is enhanced by a low LOD for TP, providing a strategic means to determine the presence of cacao in Late Classic ceramic vessels.

Imbued with sumptuary characteristics based on pictorial and textual information on Late Classic Maya vases (11, 12, 68), cacao has been set apart from “common” agricultural products without deeper inquiry. Collections from the El Pilar area include example vessel forms collected as civic and residential belongings. This diversity of contexts and vessels affords an important opportunity to test and assess significance of cacao consumption.

Our identification of the diagnostic biomarker TP provides the basis for recognizing cacao residues and raises critical questions for the Maya case. Our results underscore the need to evaluate assumptions and moderate judgments when evidence is incomplete. The suggestion that the use of cacao is restricted to or used exclusively by the elite must be set aside. There is no doubt that cacao was a precious commodity, described as a food from the gods, and our establishment of cacao in civic and residential settings indicates that cultural practices using this treasured agricultural commodity were essential to Late Classic Maya identities. It is now time to give farmers and cooks who nurtured and prepared cacao the attention they merit (69), “on the level with the priests and kings” (70).

## Methods

### Extraction.

Building on the experience of earlier studies, we use the burr-and-grind method that is destructive but minimizes potential signal from contamination, which could for example have occurred in handling and storage of the objects (5, 20, 55, 56, 71, 72). For each sample, a surface layer was ground off and a 1-cm^3^ piece was removed and ground into a fine powder. Then, 300 mg of the sample powder was weighed out for each replicate and extracted twice. Finally, 3 mL of ethanol was added to each 300-mg sample and was heated at 70 °C for 30 min. The solvent was pipetted off, filtered, and placed in a clean vial. The procedure was repeated and extracts were combined, heated gently at 50 °C, and reduced to roughly 0.5 mL.

Of the 0.5 mL of concentrated sample, 200 µL was added to 0.05 g of powdered graphite, which was gently heated again to fully dry the graphite sample mixture. The resulting material was spread onto double-sided carbon tape attached to a bar of graphite. To avoid cross-contamination among samples, each bar contained only replicates of the same sample.

### Laser MS.

L2MS offers a low LOD for TP, provides a high degree of certainty when distinguishing between isomers, such as TP and TB, and allows for accurate quantification of the ratios of the three molecules of TB, C, and TP. Owens et al. (21) validated the application of this technique in the identification of cacao in Maya pottery. L2MS has also been used for the identification of polycyclic aromatic hydrocarbons in soot residue on archaeological sherds and detection of trace material in meteorites (73) as well as other applications (74–78).

L2MS is a four-stage process, schematically depicted in *SI Appendix*, Fig. S3, consisting of: 1) laser desorption (LD), 2) jet cooling (JC), 3) resonance enhanced multiphoton ionization (REMPI), and 4) MS.

#### Laser desorption.

The sample is mixed with graphite and mounted on top of a translating substrate from which the analyte is desorbed by a focused Nd:YAG laser pulse (1,064 nm, 1 mJ, 8-ns pulse width). LD vaporizes the molecules intact, permitting detection at the parent mass peak. This LD technique has been used to successfully desorb a large variety of neutral molecules intact ranging from hydrocarbons to polymers with masses up to 8,000 Da (79–84).

#### Jet cooling.

Desorption occurs directly in front of a pulsed valve (8-atm backing pressure, 30-µs pulse width), which produces a molecular beam of argon that entrains the desorbed molecules and cools them to ∼20 °K. This low temperature permits very high-resolution spectroscopy in combination with fragment-free MS.

#### REMPI.

The ionization process is of the cold gas molecules through a two-part, two-photon process. The first tunable photon resonantly excites the molecule in a vibronic transition and the second photon ionizes the excited molecule. At the low temperatures in this technique (circa 20 °K), the optical spectra are highly and uniquely resolved for each molecule. As a result, it is possible to selectively ionize and detect a specific molecule from a complex sample. *SI Appendix*, Fig. S4 shows REMPI spectra for the three methylxanthines in this study.

Both photons are produced by an EKSPLA PL2251 Nd:YAG laser system (30-ps pulse duration). The excitation photon is produced by an optical parametric generator which converts 355-nm light into tunable UV pulses (80 to 120 uJ per pulse). The 1,064-nm and 532-nm pulses from the Nd:YAG are combined to harmonically generate 213-nm light (0.2 mJ). This 213-nm light is used for the ionization completing this step.

#### Mass spectrometry.

The final step is the detection of the ions by a time-of-flight mass spectrometer (74, 75, 79, 85). By using both wavelength and mass selectivity simultaneously, this technique can clearly distinguish between isomers: that is, compounds with different structures and the same mass (73) or tautomers (86, 87), such as TB and TP.

### Ratio Measurements.

We can quantify the ratio of the three biomarkers by using two different resonant wavelengths to simultaneously excite C and TB or C and TP, each at their own resonant wavelength (281.05, 281.6, and 280.65 nm for C, TB, and TP, respectively). Two lasers are used concurrently, one exciting C and the other exciting either TB or TP. At the same time, a third laser at 213 nm ionizes all excited molecules.

The ions of each ratio pair are separated in the mass spectrum and their mass peaks are compared to obtain the ratios. TB and TP have the same mass so their ratios are obtained indirectly by comparing the other two ratios. The main source of signal variations in L2MS is the LD step, so measuring two compounds simultaneously in the same LD sequence greatly improves the S/N in the measurement of their ratios.

Each replicate on the bar was measured twice, once for the C/TB pair and once for the C/TP pair. In each measurement, the two wavelengths stay constant while the sample is moved. Each measurement of a sample involves 12 iterations of measurement, each iteration averaging 40 laser shots.* The 12 iterations of measurement are also averaged to obtain one ratio and one signal intensity for each molecule. This is done for each of the three replicates of the extraction.

Signal represents integrated mass peaks with the background subtracted. Background is determined in two ways: 1) in an area of the mass spectrum where no mass is detected and 2) by measurements with the desorption laser blocked.

## Supplementary Material

Supplementary File

## Data Availability

The data produced in this project are in the form of scientific reports and raw data on which the reports are based. The raw data consist of spectra as a function of mass, wavelength and time, and computational data. Ceramic samples were selected on the basis of archaeological site location by reference to catalog numbers registered in a digital database on the archaeological collections of the BRASS/El Pilar project of the Institute of Social Behavior and Economic Research/MesoAmerican Research Center, UCSB. Selected ceramic samples were subjected to the analytical procedures at the UCSB Chemistry laboratory. Spectra were recorded in our UCSB Chemistry laboratory by dedicated, LabView-based, software, which we developed in house for interfacing the experimental apparatus with the computers that collect the data. We have built in options for converting and exporting the spectra in more common formats that can be read by spreadsheet or commonly used graphing software. We therefore make all of our spectra available in Excel or Origin compatible files. The computational data are routinely produced in formats that are common for such data, such as output files compatible with Gaussian and accessible with popular computational graphics packages, such as ChemCraft or Molden. Published material will be deposited in the University of California open access repository, according to the University of California open access policy. We readily share any of our data with anyone who requests them, we will also make use of our research website to emphasize their availability and to provide links to data whenever possible. Furthermore, our data will be available through the Dryad data repository Most data will be available as public domain data for fair use. We archive our data with Dryad, a self-archiving, disciplinary agnostic and CoreTrustSeal-certified data repository, which provides a number of features, including: Integration with ORCID (researcher ID) profiles; automatic persistent identifiers (DOIs) assignment to datasets; metrics (views, downloads, and citation); curation support (locally and from CDL); indexing on Thomson-Reuters Data Citation Index, Scopus, and Google Dataset Search; and integration with over 100 publishers’ workflow. Dryad is made available through the UCSB library (89).

## Footnotes

^*^A shot refers to one sequence of desorption, JC, ionization, and MS.
