# Supplementary material for: New light on the use of Theobroma cacao by Late Classic Maya
Source: Proc Natl Acad Sci U S A. 2022 Sep 26;119(40):e2121821119. doi: 10.1073/pnas.2121821119 (PMC9546560; doi:10.1073/pnas.2121821119)
Supplement: Supplementary File [file pnas.2121821119.sapp.pdf]

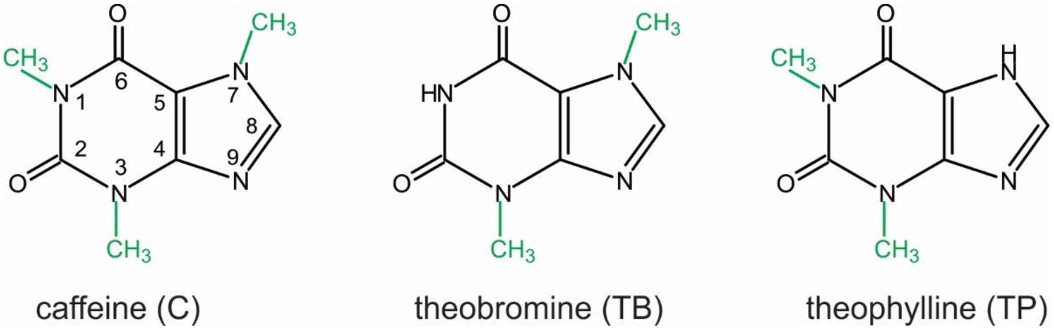

Figure S1: The three methylxanthine biomarkers used to identify Cacao.

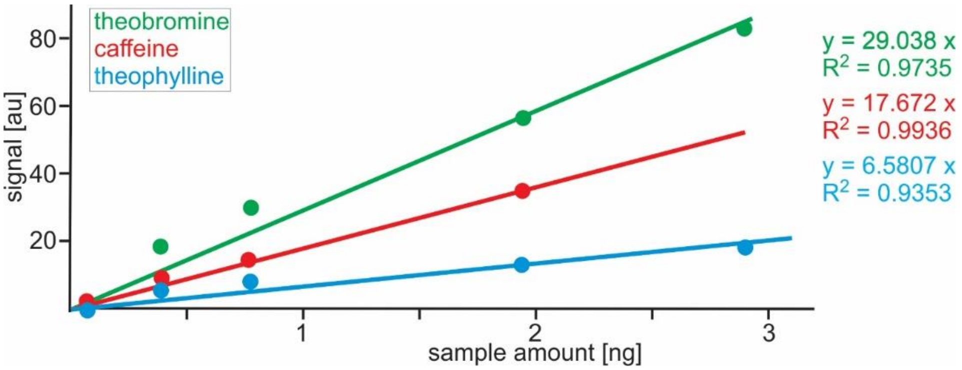

Figure S2: Calibration curves for TB (green), C (red), and TP (blue).

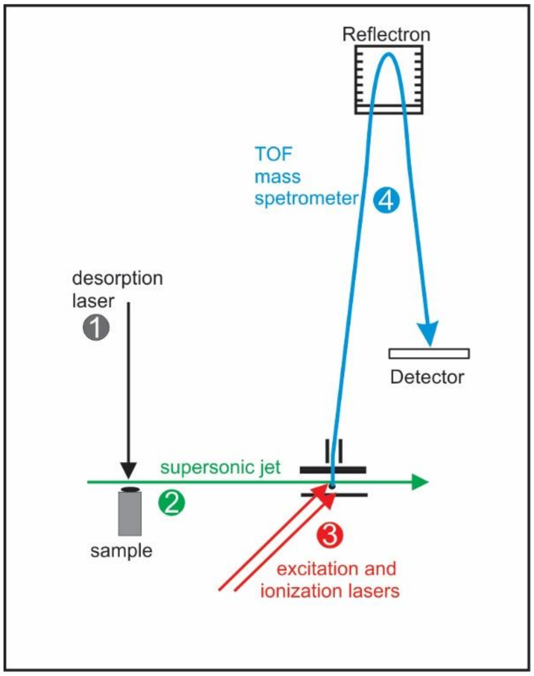

Figure S3- Diagram of the L2MS process. This technique is composed of four steps- (1) laser desorption (black arrow), (2) jet cooling (green arrow), (3) resonance enhanced multiphoton ionization (red arrows), and (4) mass spectrometry (blue arrow).

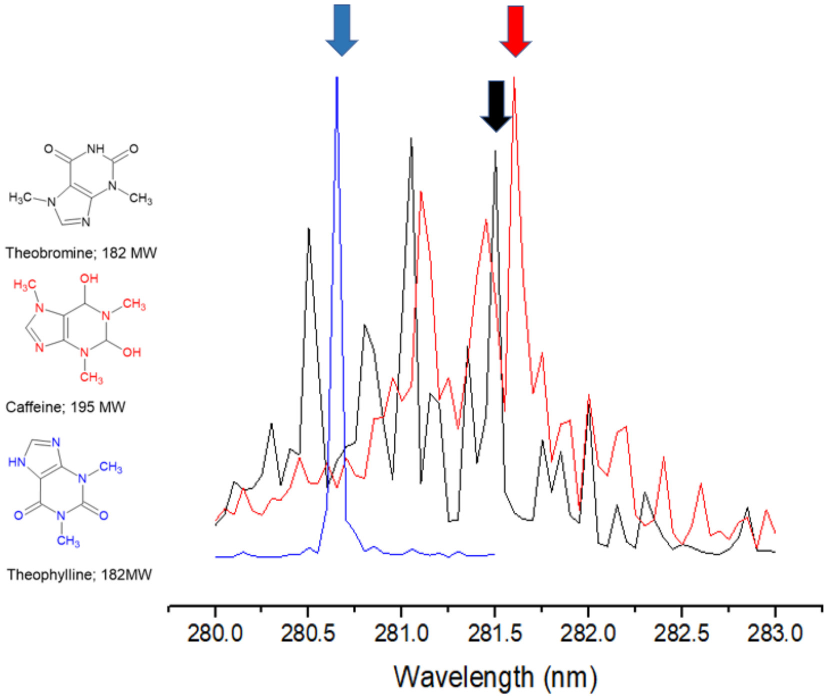

Figure S4: Resonance Enhanced Multi-Photon Ionization spectra of theobromine (black), theophylline (blue), and caffeine (red), shows that the absorption spectra are unique for the three molecules. The molecular structure of the three molecules are shown on the left, as well as the molecular weight. The arrows indicate the resonant wavelengths used in this study for the detection of each biomarker.

Table S1: *Geographic Distribution of Samples*

Site Type	Valley	Foothills	Uplands	Total
Residential Unit	12	17	17	46
Civic Center	1	2	5	8
Total	13	19	22	54

Table S2*: Summary Vessel Samples and Positive Determination for Cacao*

Form	Sample	Positive	Percent
Vases	40	26	65%
Bowls	4	1	25%
Jars	6	1	17%
Plates	4	2	50%
Total	54	30	56%

Table S3: *Distribution of Total to Samples with Cacao*

Site Type	Valley	Foothills	Uplands	Total	Percent
Residential Unit	12/7	17/13	17/7	46/27	58%
Civic Center*	1/1	2/1	5/1	8/3	38%
Total	13/8	19/14	22/7	54/30	56%

*Emphasized plates, jars, bowls
